# Impact of Cannabidiol and Its Synthetic Derivative on Fentanyl Consumption and Fentanyl-Induced Neurometabolic Alterations in Mice

**DOI:** 10.3390/molecules31193463

**Published:** 2026-09-28

**Authors:** Dalal A. AlKhelb, Jomana M. Alahmadi, Maisa S. Alqarni, Syed Rizwan Ahamad, Alaa Alnoor Alameen, Ahmed Altuwaijri, Shakir D. AlSharari, Fawaz Alasmari

**Affiliations:** 1 Department of Pharmacology and Toxicology, College of Pharmacy, King Saud University, Riyadh 11451, Saudi Arabia; 442204124@ksu.edu.sa (J.M.A.); 442202630@ksu.edu.sa (M.S.A.); aalameen1.c@ksu.edu.sa (A.A.A.); aaltuwaijri@ksu.edu.sa (A.A.); sdalsharari@ksu.edu.sa (S.D.A.); ffalasmari@ksu.edu.sa (F.A.); 2 Department of Pharmaceutical Chemistry, College of Pharmacy, King Saud University, Riyadh 11451, Saudi Arabia; srahamad@ksu.edu.sa

**Keywords:** fentanyl, cannabidiol, neurotoxicity, metabolomic, two-bottle choice, opioids

## Abstract

Background: Opioid use disorder (OUD) remains a critical global health challenge, with highly potent synthetic opioids like fentanyl contributing to overdose deaths. The available treatments have limitations. The endocannabinoid system has emerged steadily as a therapeutic target for the opioid crisis due to its modulatory effects on opioid reward and withdrawal and its involvement in regulating inflammation. These studies investigate the potential of cannabidiol (CBD) and its synthetic derivative (L-1β IN-1) in mitigating fentanyl effects. Methods: C57BL/6 male mice were used in several behavioral assays: two-bottle choice (TBC) to measure fentanyl reward/seeking behaviors, hot plate to assess antinociception, and marble burying to evaluate compulsive/adverse effects. Additionally, a neurometabolic study was conducted to evaluate the impact of chronic high doses of fentanyl alone and in combination with CBD or L-1β-IN-1 on brain metabolic signatures. Results: The mice consistently preferred to drink fentanyl, and i.p. injections of 10 mg/kg CBD or 5–25 mg/kg of L-1β-IN-1 decreased the fentanyl preference scores to values below 0.5. The antinociceptive effects of pretreatment groups showed a trend of enhanced analgesic effects in the hot plate assay. However, they showed no significant changes in the marble-burying assay, suggesting that pretreatment with these compounds lacks acute stereotypic effects. The Fentanyl metabolomics revealed robust metabolic activation with fentanyl alone, while CBD or L-1β-IN-1 showed different profiles in modulating these fentanyl-induced metabolic signatures. Conclusions: These studies provide evidence about the in vivo behavioral and neurometabolic profiles of fentanyl after pretreatment with CBD and synthetic CBD, presenting them as possible pharmacological tools for OUD research.

## 1. Introduction

Over the past decade, there has been a shift in the opioid crisis, moving from prescription-opioid misuse to a third-wave crisis dominated by illicitly manufactured synthetic opioids [1]. While data from the CDC in 2025 indicated a decline in overall overdose deaths, fentanyl remains the primary driver of mortality, involved in ~70% of all drug-related deaths [2]. In addition, the fentanyl crisis remains on the rise globally without adequate treatment options [3,4]. Methadone and Buprenorphine are effective pharmacotherapies but have a risk of relapse and misuse potential [5,6]. Importantly, these treatments interact at the receptor level and aim mainly to suppress withdrawal symptoms and reduce cravings by modulating activation at the µ-opioid receptor [7]. However, their efficacy in improving the underlying metabolic dysregulation, which is a major contributor to opioid-induced chronic neurobehavioral changes, remains limited [8].

Evidence suggests that the transition from recreational drug use to addiction is mediated by neuroinflammatory processes within the central nervous system (CNS) [9,10]. For example, chronic opioid exposure activates microglia and astrocytes by increasing proinflammatory cytokine levels, including tumor necrosis factor-alpha (TNF-α), interleukin-6 (IL-6), and interleukin-1beta (IL-1β) [8,11,12]. Moreover, the behavioral maladaptation associated with opioid use appeared to be driven by IL-1β neuroinflammatory signaling that disrupts synaptic plasticity and alters reward circuitry, creating a persistent state of neural distress [11,13]. Additionally, brain metabolomic studies indicated that chronic fentanyl exposure leads to profound metabolic reprogramming by showing a clear shift in energy utilization and the disruption of key neurotransmitter pathways. Particularly, a compensatory upregulation of carbohydrate metabolism to meet the high energy demand from the persistent pro-inflammatory states that drive oxidative stress and neurotoxicity was reported after chronic fentanyl exposure [12,14]. Despite these findings, there is limited data about the efficacy of using synthetic CBD with potent pro-inflammatory cytokine inhibitors as a therapeutic strategy in modulating opioid adverse effects.

The longstanding data on cannabinoids’ therapeutic potential for inflammation-related conditions [15,16], combined with their interaction with opioids, make them a promising drug target to modulate opioid adverse effects [17,18]. Mechanistic studies show that cannabinoids can modulate opioid effects either by direct receptor interactions, alterations in endogenous peptide release, or shared signaling cascades [17,19]. Moreover, the role of neuroinflammation in addiction has gained attention, with cannabinoids recognized as potent immunomodulators of these effects [20,21,22]. Studies have shown that cannabinoids can inhibit cytokine production and suppress microglial activation, which play key roles in addiction and neurotoxicity [16,23]. Additionally, evidence suggests that orthosteric CB_1_ antagonists reduce the rewarding effects of opioids, but CBD, as a non-psychoactive natural cannabinoid receptor modulator, offers additional neuroprotective benefits by modulating receptor activity without completely blocking its function [18,19,24]. The present study was designed to characterize the behavioral and neurometabolic correlates of the impact of CBD and a structurally similar derivative (L-1β-IN-1) on fentanyl effects. Therefore, we addressed the question of whether CBD and L-1β-IN-1 have different pharmacological profiles in mitigating fentanyl reward/seeking behaviors, antinociception, and acute compulsive/adverse effects. Additionally, we used untargeted brain metabolomic analysis to evaluate whether CBD and L-1β-IN-1 are associated with differential neurometabolic signatures against chronic fentanyl exposure.

## 2. Results

### 2.1. Effects of CBD or L-1β-IN-1 on Fentanyl Consumption in the Two-Bottle Choice

Water consumption was maintained at ~0.12 mL during the posttreatment short-access sessions. Fentanyl increased this volume to 0.27 mL, and pretreatment with CBD reduced it to 0.1 mL. Similarly, pretreatment with either 5 or 25 mg/kg L-1β-IN-1 reduced water consumption to 0.04 and 0.07 mL, respectively. However, 4 of the mice in the 25 mg/kg group were found dead after the first fentanyl session.

**Figure 1:**
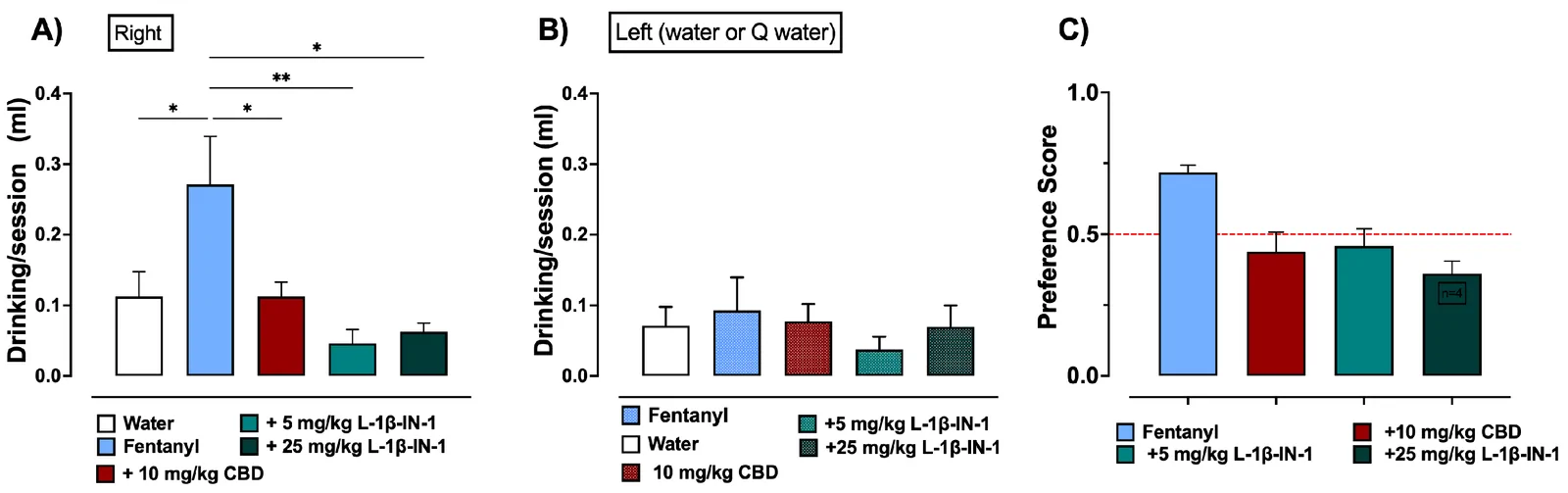
Fentanyl seeking and preference behaviors in TBC. **A**) Volume of drinking of water or fentanyl from the right bottle choice after pretreatment sessions with i.p. injections of CBD or L-1β-IN-1. **B**) Volume of drinking of water or Q water from the left bottle choice after pretreatment sessions with i.p. injections of CBD or L-1β-IN-1. **C**) Fentanyl preference score across the groups. The dashed red horizontal line indicates that a score of 0.5 represents equal preference among bottle choices. Error bars represent S.E.M. Each data point represents all subjects tested (*n* = 6–8 mice) at each dose except 25 mg/kg L-1β-IN-1, *n* = 4; the other 4 were lost after the pretreatment session. * *p* < 0.05, ** *p* < 0.01.

To confirm that these reductions in drinking are not due to behavioral disruption, the volumes from non-fentanyl bottle choice have a consistent volume of drinking ~0.07 mL across all groups (Figure 1A,B). ANOVA analysis reported a statistically significant difference among the group means (F_(4, 30)_ = 5.260; *p* < 0.01). Post hoc evaluation using Tukey’s multiple comparisons test showed that Fentanyl significantly increased the baseline values compared to the control group (*p* < 0.05). Conversely, no significant differences were observed when comparing the Water control group to the 10 mg CBD group, the 5 mg/kg L-1β-IN-1 group, or the 25 mg/kg L-1β-IN-1 group (*p* > 0.05).

The fentanyl preference score, as a primary measure of reward behavior, showed that in 6-h water vs. fentanyl choice sessions, the mice consistently preferred to drink 0.03 mg/mL fentanyl with a preference score above 0.5. Pretreatment with i.p. injections of 10 mg/kg CBD decreased the fentanyl preference scores to values below 0.5. In a similar manner, pretreatment with 5 or 25 mg/kg of L-1β-IN-1 also decreased the fentanyl preference < 0.5 in a dose-dependent manner (Figure 1C).

### 2.2. Effects of CBD or L-1β-IN-1 on Chronic Fentanyl Consumption in the Hot Plate Assay

**Figure 2:**
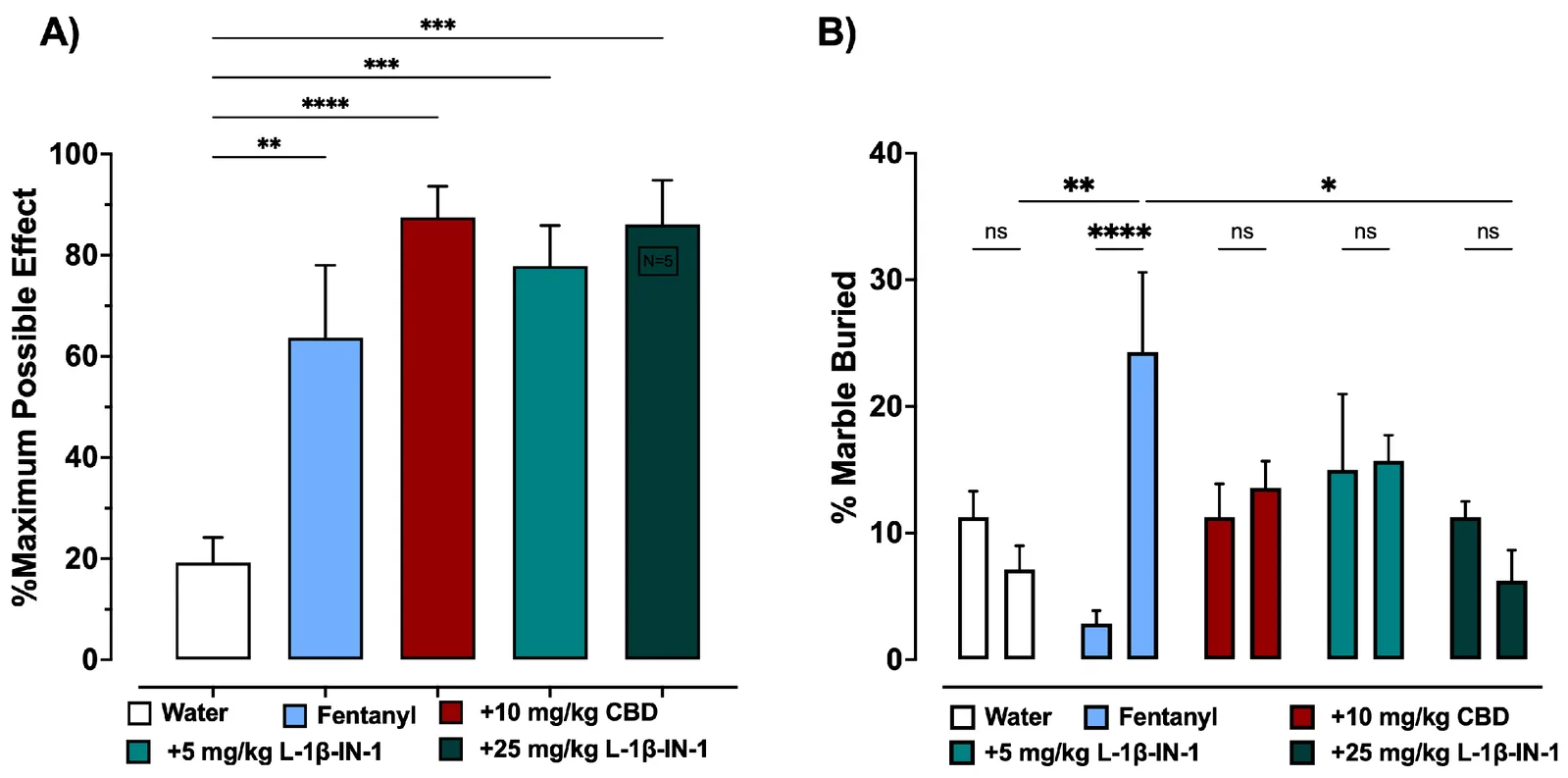
Effects of CBD or L-1β-IN-1 on fentanyl-induced behavioral changes. **A**) Antinociceptive effects of fentanyl alone and after pretreatment with CBD or L-1β-IN-1 in the hot plate assay. **B**) Percentage of marbles buried %MB at baseline (right) and post TBC experiments. Error bars represent S.E.M. Each data point represents all subjects tested (*n* = 6–8 mice) at each dose except 25 mg/kg L-1β-IN-1, *n* = 4. * *p* < 0.05, ** *p* < 0.01, *** *p* < 0.001, **** *p* < 0.0001; ns, not significant (*p* ≥ 0.05).

The average %MPE for the control (water) group was 19%. Fentanyl produced 64% after the last session of TBC, whereas pretreatment with 10 mg/kg CBD, 5 mg/kg, or 25 mg/kg of L-1β-IN-1 produced 87.5%, 78%, and 86%MPE, respectively (Figure 2A). All treatment groups produced a significant antinociceptive effect compared to the control group (F_(4, 29)_ = 8.67; *p* = 0.0001). Dunnett’s multiple comparisons test between water vs. fentanyl, CBD, and L-1β-IN-1 was significantly different. However, the comparisons between fentanyl vs. pretreatments revealed no statistical changes, indicating that all three groups have good antinociceptive effects in this assay.

### 2.3. Effects of CBD or L-1β-IN-1 Treatments on Chronic Fentanyl Consumption in the Marble-Burying Test

The average %MB for baseline in all the experiment groups was ~7–15%, whereas %MB after the last session of TBC indicates 7% of MB in the water group, 24.3% of MB in the fentanyl group, 13.6% in the group pretreated with 10 mg/kg CBD, and 15.7% and 6.25% in the group pretreated with either 5 mg/kg, or 25 mg/kg of L-1β-IN-1, respectively (Figure 2B). Two-way ANOVA showed a statistical difference between the groups (F_(4, 67)_ = 4.3; *p* < 0.01), and Tukey’s multiple comparisons test showed that only the fentanyl group differed significantly from its baseline value (*p* < 0.0001).

### 2.4. The Effects of CBD or L-1β-IN-1 in Chronic High Doses of Fentanyl Administration in Brain Metabolomics Analysis

- Differential Expression of Metabolites Across Treatments

**Figure 3:**
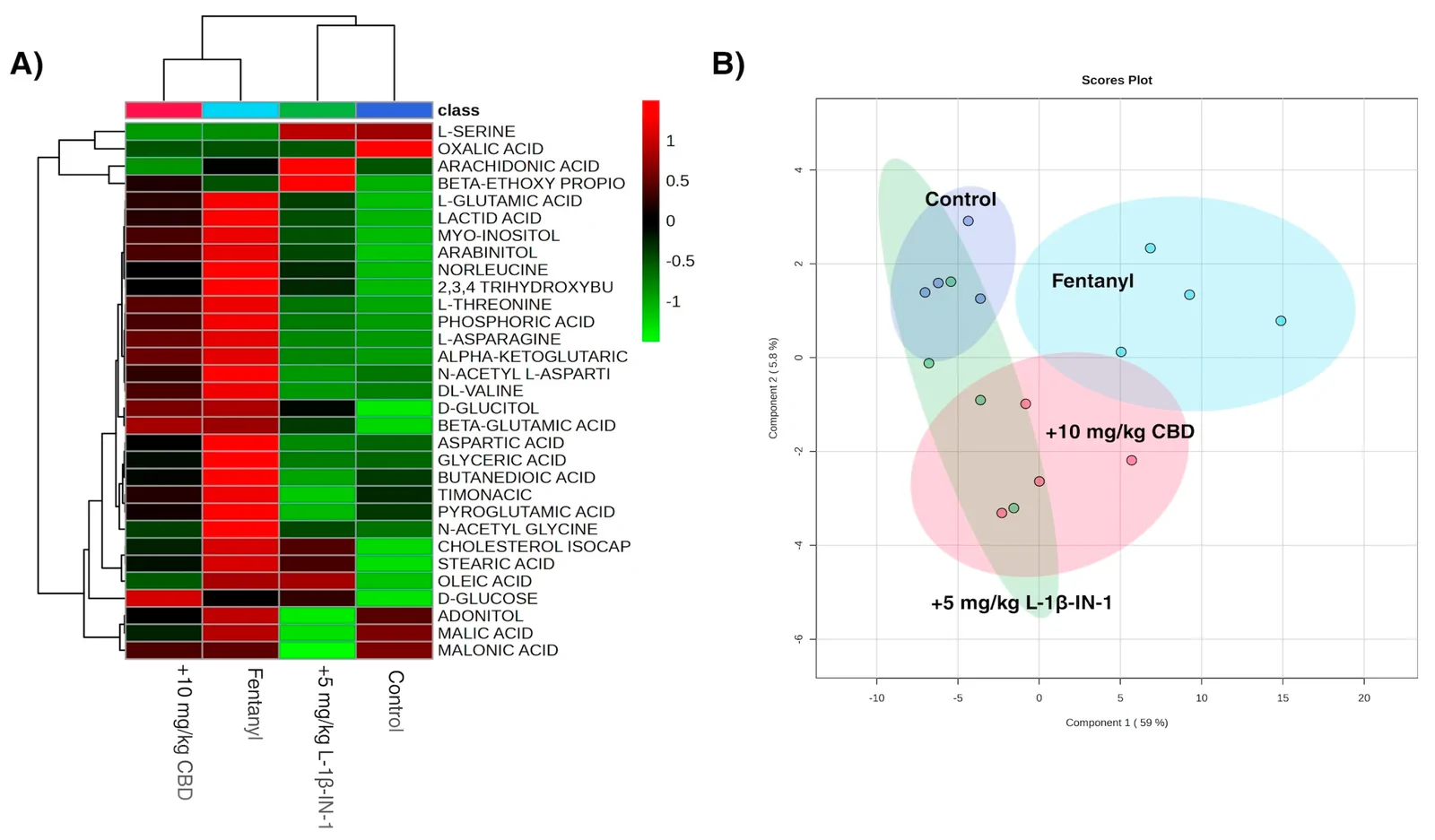
Differential Expression of Metabolites Across Treatments. **A**) The heatmap displays the relative levels of detected metabolites in the control, fentanyl group, fentanyl + 10 mg/kg CBD, and fentanyl + L-1β-IN-1 groups. Rows represent a specific metabolite, and each column corresponds to experimental groups. The color scale ranges from green, indicating lower metabolite levels, to red, indicating higher metabolite levels. **B**) Sparse Partial Least Squares Discriminant Analysis (sPLS-DA) score plot illustrates the separation of metabolite profiles between the control group vs. the fentanyl groups with or without pretreatment. Purple circles (dark blue cluster) are the control group, light blue circles (light blue cluster) are the fentanyl overdose group, red circles (pink cluster) are the fentanyl + CBD group, and green circles (green cluster) are the fentanyl + L-1β-IN-1 group samples (*n* = at least 4 hemispheres in each group).

The heatmap in Figure 3A represents the data derived from the left hemisphere’s metabolomic analysis to identify the overall patterns in the abundance of the brain metabolites following high-dose and chronic fentanyl exposure. The rows in the matrix show changes in the metabolites, whereas the columns illustrate the experimental groups. The intensity of the color scale reflects the metabolite abundance, in which darker green represents a very low abundance of the metabolite and dark red represents an extremely high abundance of the metabolite. Fentanyl exposure yielded a prominent change in the metabolomics signature, with most of the energy redox, neurochemistry (glutamate), inflammation, and osmoregulation-related metabolites being upregulated. Pretreatment with 10 mg/kg CBD showed a specific normalization effect in several upregulated signatures, including L-Serine and Oxalic Acid. In the 5 mg/kg L-1β-IN-1 pretreated group, the data showed broader normalization of the spiked metabolites. The pairwise Pearson correlation matrices for all 31 detected metabolites, highlighting their significance, are shown in Supplementary Figure S1.

Tukey’s pairwise comparisons on the detected metabolites showed that L-1β-IN-1 differed significantly from fentanyl alone in 11 metabolites (35%), including both TCA cycle intermediates and the two most strongly fentanyl-elevated metabolites, myoinositol and NAA, and did not differ from control in 29 metabolites (94%). In comparison, CBD differed significantly from fentanyl in only butanedioic acid, glyceric acid, and norleucine (10% of the detected metabolites). These differential effects suggest that at the doses and sample size tested, L-1β-IN-1 has different and broader normalization profiles for fentanyl-induced metabolic signature than CBD (Supplementary Figure S2).

- Partial Least Squares Discriminant Analysis (PLS-DA)

The sPLS-DA scores plot illustrates a high degree of class discriminative power, particularly along Component 1 (59%), which effectively separates the fentanyl group, represented as a light blue cluster, from the dark blue control and the pink cluster of 10 mg/kg pretreatment with CBD or the green cluster of 5 mg/kg L-1β-IN-1 pretreatment groups (Figure 3B). The Control and 10 mg/kg CBD-pretreated groups are primarily differentiated along Component 2 (5.8%), showing a distinct and non-overlapping cluster. The 5 mg/kg L-1β-IN-1 group exhibits a unique profile with a distinct center and an elongated 95% confidence interval that overlaps the Control and CBD groups.

Multivariate analysis of the neurometabolomics revealed significant differences among the four experimental groups. PERMANOVA analysis showed a significant overall group effect (F = 9.3897, *p* = 0.005; 199 permutations), indicating that group membership accounted for approximately 70.1% of the variation in the distance matrix. Pairwise PERMANOVA identified nominal differences between the CBD and control groups (*p* = 0.020), L-1β-IN-1 and fentanyl groups (*p* = 0.020), and control and fentanyl groups (*p* = 0.035), whereas the remaining comparisons, CBD vs. fentanyl and L-1β-IN-1 vs. control, were not statistically significant (*p* > 0.05). PLS-DA permutation testing further supported the observed group discrimination, with an empirical *p*-value of 0.015 based on 2000 permutations, suggesting that the supervised separation was unlikely to have occurred by chance. In addition, sPLS-DA cross-validation showed improved classification performance with two latent components, as the classification error decreased from 43.8% with one component to 18.8% with two components, corresponding to an accuracy of approximately 81.2%. Collectively, these findings indicate overall alterations in neurometabolomic profiles and support the ability of the two-component sPLS-DA model to discriminate among the experimental groups.

- The Inter-relationships Among Brain Metabolites

**Figure 4:**
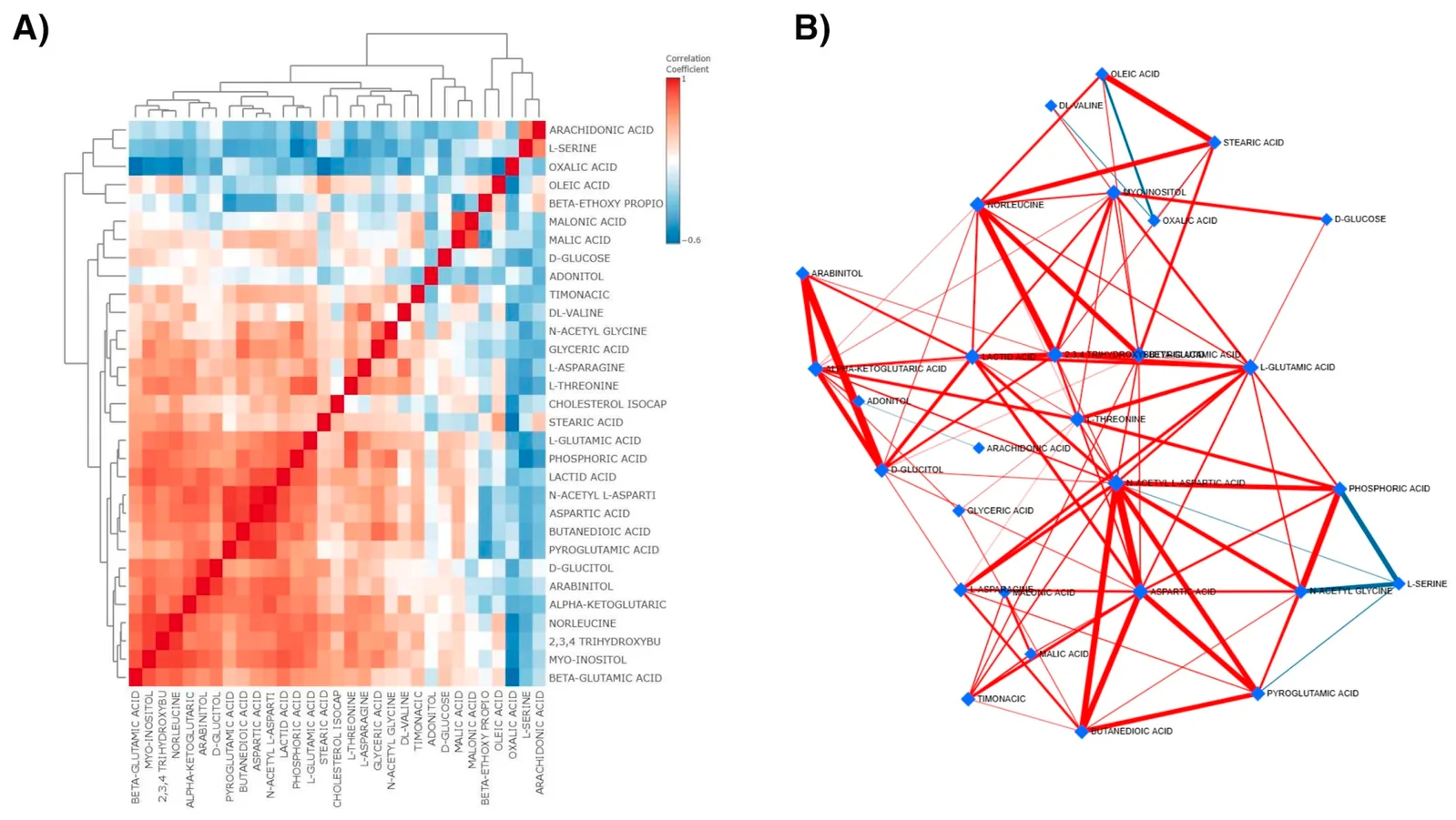
Inter-relationships Among Brain Metabolites and Network Analysis. **A**) Correlation matrix of detected brain metabolites from the left-brain hemispheres, and the color scale represents the strength and direction of pairwise correlations; red indicates a positive correlation, whereas blue indicates a negative correlation. **B**) Network analysis of disrupted metabolites in the chronic fentanyl group. The Pearson correlation between significantly altered metabolites is illustrated as nodes and edges. Each node represents a specific metabolite, and the edges connecting nodes indicate correlation between the connected metabolites. The size of each node represents a metabolite and corresponds to the magnitude of change in metabolite level, with larger nodes representing greater alterations. The color of the edges represents the type of correlation between metabolites: red edges are positive correlations, whereas blue edges are negative correlations.

The correlation matrix of metabolites (Figure 4A) represents a list of the detected metabolites from the left-brain hemispheres, and the color scale represents the strength and direction of pairwise correlations, where red indicates a positive correlation whereas blue indicates a negative correlation. These data show a strong diagonal of red signature, indicating ideal self-correlation for each metabolite, and show distinct clustering patterns across the matrix, observed as a large block of warm red to orange colors, which suggest that these metabolites are positively correlated, consistent with but not proof of shared regulation or involvement in related metabolic pathways. Importantly, in this dataset, there is a strong positive correlation among amino acids and organic acids, with all pairwise comparisons among Glutamic Acid, Aspartic Acid, and Phosphoric Acid, N-Acetyl-L-Aspartic acid (NAA), Alpha-Ketoglutaric acid, and butanedioic acid reaching statistical significance (*p* < 0.001), including a particularly strong correlation between Aspartic Acid and NAA (*p* < 0.0001; Supplementary Figure S1), suggesting associated co-regulation within the Tricarboxylic Acid cycle (TCA) and Glutamate metabolism pathways.

- Network Interactivity Analysis

The metabolite network shows a highly interconnected metabolic system with several significant associations among the detected metabolites (Figure 4B). The majority of the metabolites are linked through dense connections, indicating strong coordination across multiple metabolic pathways. Specifically, myo-inositol, 2,3,4-trihydroxybutyric acid, phosphoric acid, and aspartic acid show the highest network connectivity, with 19–20 significant correlates having a *p*-value < 0.05, emerging as the central metabolites, with several other metabolites, including L-glutamic acid and lactic acid, N-acetyl-L-aspartic acid, alpha-ketoglutaric acid, and butanedioic acid also densely connected (17–18 metabolites). The network also shows the presence of distinct clusters of metabolites, such as organic acids and amino acids, which are tightly grouped and might relate to specific biological pathways. The predominance of strong positive associations indicated by thick red edges suggests that many metabolites change in a coordinated manner, possibly due to shared biochemical pathways or common regulatory mechanisms. On the other hand, the fewer blue edges indicate weaker or negative relationships, suggesting selective or opposing metabolic interactions. Overall, these results indicate a complex and integrated metabolic network, with key hub metabolites potentially serving as important biomarkers or regulators for fentanyl-driven brain metabolic changes. We note that this network was derived from correlational co-abundance data within the fentanyl group only and reflects statistical association rather than a tested causal or regulatory relationship across these metabolites.

- Effects of CBD or L-1β-IN-1 on Fentanyl-Driven Brain Metabolite Fluctuations

**Figure 5:**
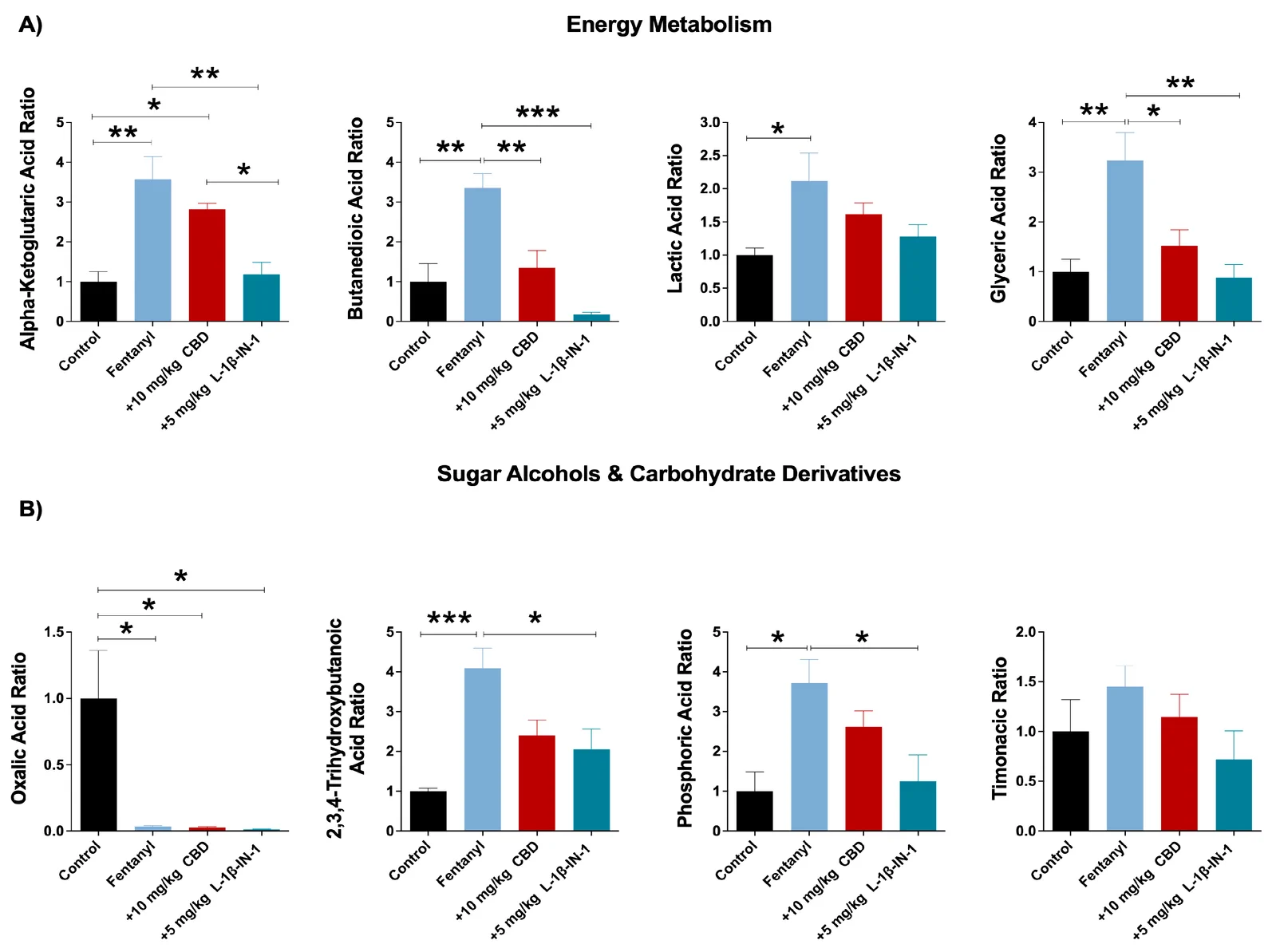
Effects of CBD or L-1β-IN-1 on fentanyl-driven brain metabolite fluctuations in energy and carbohydrate pathways. **A**) Relative abundance ratios of key energy metabolism metabolites. **B**) Relative abundance ratios of key sugar alcohols and carbohydrate derivatives. The bar graphs represent the normalized mean ratio of metabolite peak areas across the experimental groups, with the control group serving as the baseline (ratio = 1). Bars represent mean ± SEM. Statistical significance is indicated as * *p* < 0.05, ** *p* < 0.01, *** *p* < 0.001.

**Figure 6:**
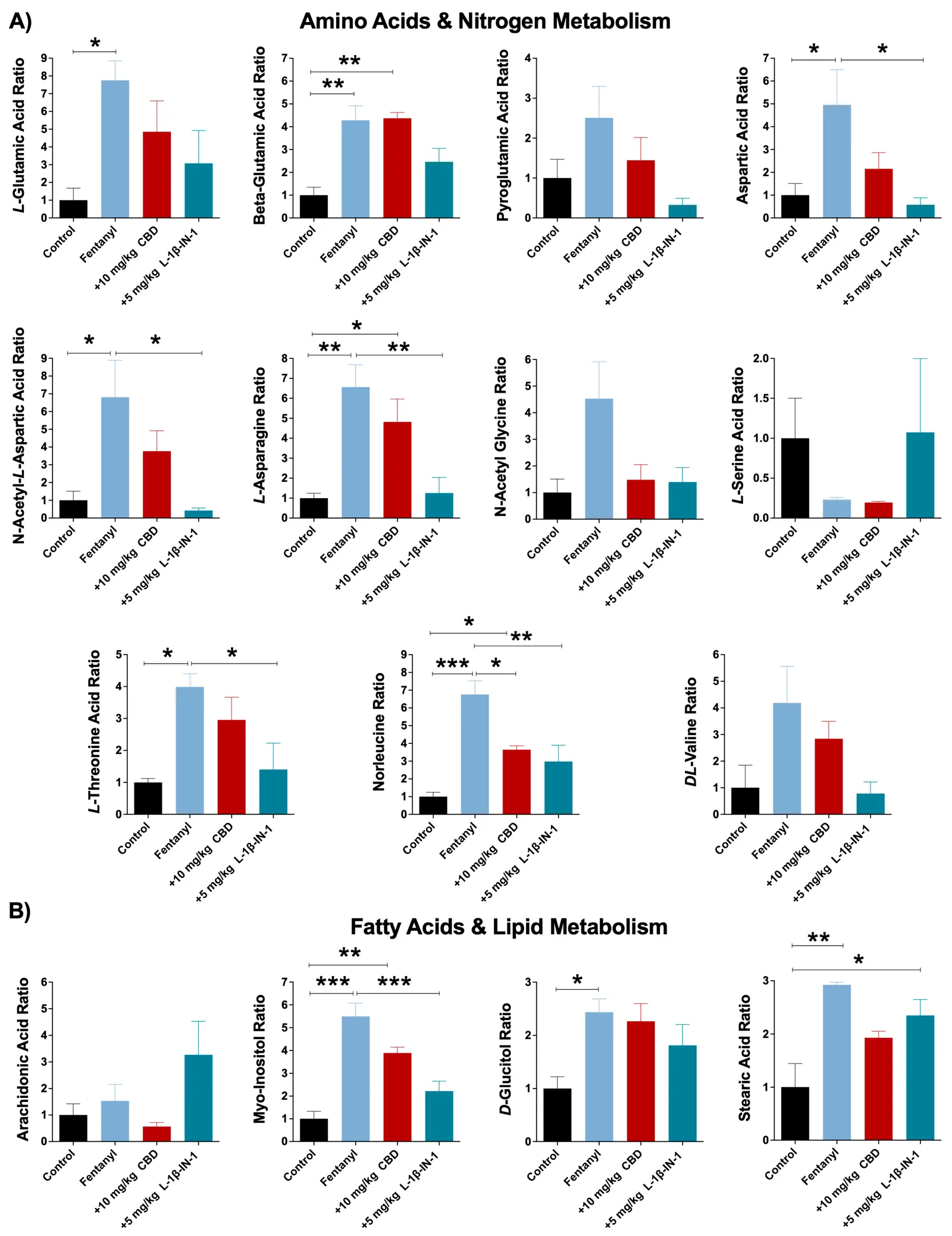
Effects of CBD or L-1β-IN-1 on fentanyl-driven brain metabolite fluctuations in amino acids and lipid pathways. **A**) Relative abundance ratios of key amino acids and nitrogen metabolites. **B**) Relative abundance ratios of key fatty acids and lipid metabolism. The bar graphs represent the normalized mean ratio of metabolite peak areas across the experimental groups, with the control group serving as the baseline (ratio = 1). Bars represent mean ± SEM. Statistical significance is indicated as * *p* < 0.05, ** *p* < 0.01, *** *p* < 0.001.

The non-targeted metabolomic analysis identified 31 metabolites across the four study groups: Control, Fentanyl, and pretreatment groups with 10 mg/kg CBD or 5 mg/kg L-1β-IN-1. ANOVA analysis revealed that 18 metabolites differed significantly (*p* < 0.05) across the study groups. Tukey’s HSD post hoc tests further analyzed specific pairwise differences among these significant metabolites (*p* < 0.05; Figure 5 and Figure 6). Fentanyl produces a significant change in the peak ratios in several metabolites, including a ~2–3-fold increase in the lactic acid, D-Glucitol, and stearic acid ratios compared to the control, a ~4-fold increase in the Phosphoric acid, L-threonine, Alpha-ketoglutaric acid ratio, 2,3,4-Trihydroxybutanoic acid, and Beta-glutamic acid ratios. Also, there is a 3-fold increase in the Butanedioic acid and Glyceric acid ratios. A ~5-fold increase in the aspartic acid ratio, ~6–7-fold increase in L-asparagine, N-Acetyl-L-Aspartic acid, Norleucine, Myo-Inositol, and ~8-fold increase in the L-glutamic acid ratio. The Oxalic acid ratio was significantly reduced in the fentanyl group. Oxalic acid, Phosphoric acid, Butanedioic acid, L-Threonine, Glyceric acid, Aspartic acid, Beta-glutamic acid, Alpha-ketoglutaric acid, L-asparagine, 2,3,4-Trihydroxybutanoic acid, N-Acetyl-L-Aspartic acid, Norleucine, Stearic acid, and Myo-inositol exhibited significant differences across multiple group comparisons. In addition, several fentanyl vs. CBD comparisons based on normalized peak ratios were insignificant (*p* > 0.05), including alpha-ketoglutaric acid, lactic acid, L-glutamic acid, N-Acetyl-L-Aspartic Acid, L-Asparagine, L-threonine, Myo-inositol, D-Glucitol, and stearic acid. In contrast, the fentanyl vs. L-1β-IN-1 comparisons reached statistical significance in most of these metabolites, including butanedioic acid, glyceric acid, Myo-inositol, and L-Asparagine (*p* < 0.001 or *p* < 0.0001). Supplementary Table S1 includes pairwise statistical analysis for all the listed metabolites.

### 2.5. The Effects of CBD or L-1β-IN-1 in Chronic High Doses of Fentanyl Administration in Open Field Studies

**Figure 7:**
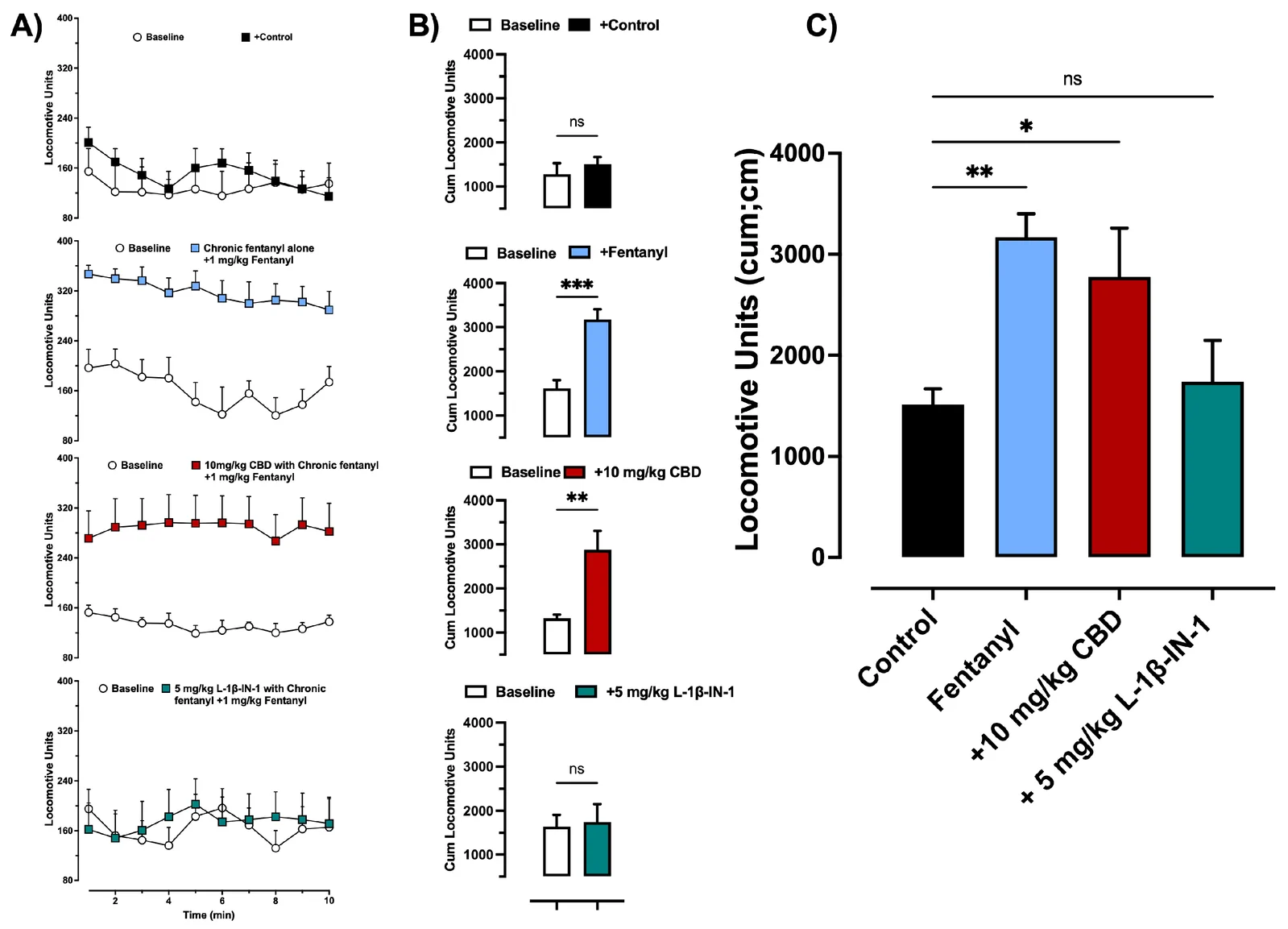
Effects of CBD or L-1β-IN-1 in Chronic High Doses of Fentanyl Administration in Open Field Studies. **A**) Time course (left) of locomotor activity and 10-min cumulative activity for baselines and after chronic and high-dose fentanyl administration. **B**) Bar graphs show the cumulative change in locomotor activity between groups after chronic and high-dose fentanyl challenge with and without CBD or L-1β-IN-1. Error bars represent S.E.M. **C**) Comparison of cumulative locomotor activity between groups after treatment (*n* = 8 in each group). * *p* < 0.05, ** *p* < 0.01, *** *p* < 0.001, ns = not significant (*p* ≥ 0.05).

The first experiment to determine baselines showed consistent activity during the 10 min test period with no significant changes in the total locomotor counts among the tested groups: control, fentanyl, and pretreatments with 10 mg/kg CBD or 5 mg/kg L-1β-IN-1. After the daily chronic fentanyl injections, the groups were challenged with a high dose of 1 mg/kg fentanyl 10 min prior to the test. The data showed a significant ~2-fold increase in the ambulatory counts as an indication of hyperlocomotion in the fentanyl group, as well as the 10 mg/kg CBD + fentanyl group, compared to the control group. Conversely, challenging the 5 mg/kg L-1β-IN-1 group with fentanyl failed to produce a significant enhancement in the counts during the test session (Figure 7; F_(3, 27)_ = 5.81, *p* < 0.01).

## 3. Discussion

Accumulating evidence suggests the potential of the endocannabinoid system as a strategy to mitigate opioid-related adverse effects [21,25]. This study aims to investigate these effects using CBD and its synthetic derivative. We utilized L-1β-IN-1, a commercially available CBD analog and potent IL-1β inhibitor, to evaluate fentanyl behavioral and neurometabolic effects. We selected L-1β-IN-1 as a CBD-based derivative that showed a strong in vitro and in vivo IL-1β inhibitory profile; it was reported to cause a significant and more potent reduction in IL-1β plasma in LPS-induced peritonitis. It also inhibited IL-1β mRNA and protein expression compared to CBD [26]. Taking this direct evidence about L-1β-IN-1 as a strong proinflammatory cytokine inhibitor in inflammation-challenged models, we examined how L-1β-IN-1 would differentially modulate the behavioral and neurometabolic changes induced by fentanyl compared with the naturally occurring cannabinoid CBD. First, we tested whether CBD or L-1β-IN-1 would modulate the fentanyl preference in the TBC drinking paradigm. The data from these studies showed that the mice consistently preferred to drink from the fentanyl bottles, and pretreatment with CBD or L-1β-IN-1 reduced this preference score. The TBC paradigm is a well-established method for measuring voluntary preference in opioids. Several studies validated oral fentanyl and oxycodone intake in mice, suggesting that animals voluntarily drink a sufficient opioid to precipitate naloxone-induced withdrawal, confirming that the fentanyl drinking behavior is attributed to its true pharmacological rewarding effect [27,28,29]. Our data on drinking volumes from quinine-adulterated (i.e., non-fentanyl) bottles indicate stable intake compared to the water control across all groups, with no statistically significant differences among groups. These data confirm that the animals in all tested groups remained actively engaged in drinking behavior and that the reduction in preference scores after CBD or L-1β-IN-1 is unrelated to behavioral disturbances in the tested animals. Overall, our observation is consistent with several studies showing that CBD blocked the opioid-induced rewarding and seeking effects [30,31,32]. The replicated effect of L-1β-IN-1 in reducing fentanyl preference and seeking suggests that a similar chemical structure might engage the same CB receptor modulation, but further docking studies are required to confirm this speculation. Additionally, future studies are needed to address whether the reduction of preference after CBD and L-1β-IN-1 pretreatment is due to inhibiting IL-1β in fentanyl-treated animals.

Similar to fentanyl, CBD and cannabinoid agonists are known for their antinociceptive effect in preclinical and clinical data [15,33,34]. To capture whether these antinociceptive effects were observed in the animal groups of the TBC paradigm, we used the hot plate assay. A numerical increase in the fentanyl effect was observed from ~60% to ~80% in the pretreatment groups. However, these differences did not reach statistical significance; we interpret this as a trend towards potentiating fentanyl antinociceptive effects. The presented data did not systematically explore whether combinations of low doses of L-1β-IN-1 or CBD would have a leftward shift in the fentanyl antinociceptive effects, which is the key indicator for opioid-sparing effects. Nevertheless, the observed trend encourages further exploration of whether these pretreatments would alter fentanyl dose-response effects on nociception and respiratory functions. These are important studies to determine the safety of these combinations. In particular, it is important to test whether CBD and L-1β-IN-1 would also enhance the respiratory depressant effects of fentanyl. These future studies are important to confirm whether L-1β-IN-1 effects are consistent with the previously reported opioid-sparing effects with CBD and CB agonists [15,35].

Acute toxicities through behavioral/stereotypic effects are a major concern in opioid and cannabinoid preclinical studies. We utilized the marble-burying assay to test if these pretreatments might induce signs of behavioral toxicity in our TBC paradigm. Marble- burying baselines of pre-drinking behaviors were compared with post-drinking outcomes by assessing the percentage change in marbles buried after the final session. Only the fentanyl group had a significantly different result from the baseline, suggesting that fentanyl induced a trend of compulsive behaviors that might be explained either as compulsive or hyperlocomotive behaviors in this paradigm. While the data on how opioids change the %MB is limited, with some studies showing that fentanyl or morphine withdrawal increases marble-burying behavior [36,37,38], there is mounting evidence about fentanyl generally being a highly addictive substance that causes hyperlocomotion and induces anxiety-like and compulsive seeking stereotypic behaviors [39,40]. Interestingly, the changes from baseline in pretreatment groups were insignificant despite the similar intermittent exposure to fentanyl in the pretreatment groups, which suggests that CBD and L-1β-IN-1 have a modulatory effect in the marble-burying assay. Notably, the reduction in acute toxicity following L-1β-IN-1 pretreatment raises the question of whether it will have anxiolytic effects and whether the previously observed sex-dependent anxiolytic effects of CBD alone or in combination with opioids could generalize to L-1β-IN-1 [24,41,42]. However, the presented MB data should be interpreted with caution as evidence of the efficacy of CBD and L-1β-IN-1 in reducing the acute behavioral toxicity rather than a demonstrated anxiolytic effect. Future studies should determine whether these effects are sex-dependent and should employ validated specialized anxiety assays, such as the elevated plus maze in male and female mice, to directly assess the potential anxiolytic effects of L-1β-IN-1 and CBD.

An interesting finding from the above-mentioned studies was that pretreatment of 25 mg/kg L-1β-IN-1 caused death in 4 subjects. While it is hard to point out the exact reason for the deaths, we believe that this is due to an interactive effect with fentanyl rather than toxicity from the 25 mg/kg L-1β-IN-1 alone, as this was reported as an active in vivo dose on the manufacturer’s website, as well as in the pilot rodent studies showing that it is safe and effective in inflammatory pain [26]. Based on the available literature, our leading explanation is a dose-threshold pharmacokinetics interaction. Fentanyl is well documented to be metabolized by hepatic CYP3A4, and CBD, along with other structurally related cannabinoids, is an established concentration-dependent inhibitor of CYP450 isoforms including CYP3A4. Studies show that the inhibitory potency increases disproportionately at higher concentrations rather than scaling linearly with dose [43,44]. Hence, we hypothesize that L-1β-IN-1 might have similar PK to CBD and that the dose of 25 mg/kg may have crossed a concentration threshold sufficient to meaningfully inhibit fentanyl metabolism, producing elevated and potentially lethal fentanyl exposure, whereas this threshold was not reached at the lower dose. Importantly, this hypothesis is directly supported by evidence in male C57BL/6J mice showing that acute CBD administration inhibited oxycodone metabolism, producing a three-fold increase in total oxycodone exposure and a 50% increase in peak plasma concentration, indicating that CBD can meaningfully elevate systemic opioid exposure through a pharmacokinetic mechanism [45]. Although this specific interaction has not yet been characterized for fentanyl in combination with the 25 mg/kg L-1β-IN-1, it supports the possibility of a dose-dependent pharmacokinetic interaction underlying the mortality observed at the high dose of 25 mg/kg. Overall, these behavioral data build on evidence that CBD can modulate opioid-related effects by showing generalizable effects with its synthetic analogs in reducing fentanyl drinking, maintaining the antinociceptive effects, and modulating the general behavioral toxicity.

A systematic analysis of changes in brain metabolites is correlated with general behavioral outcomes from open field studies to investigate the role of CBD and its synthetic analog in modulating the complex neurobiological and behavioral interaction of chronic fentanyl administration. The findings showed that exposing mice to a high dose of fentanyl profoundly alters the metabolomic signatures in the left hemisphere by showing a robust elevation of excitatory amino acids and mitochondrial-associated metabolites, as well as organic acids. These data are consistent with previously published studies showing profound changes in brain metabolomics in specific brain regions yet further highlight the broad-spectrum neurotoxic effects of fentanyl in the whole brain hemisphere [12,14]. While these data are important to understand the global neuroprotective role of CBD and its synthetic derivative, additional metabolomic studies in selective brain regions could be beneficial to map each brain region with the behavior (e.g., nucleus accumbens and reward).

The data show that fentanyl drives metabolic stress, and pretreatment with 5 mg/kg L-1β-IN-1 normalized fentanyl induced metabolic alterations more consistently than the 10 mg/kg CBD pretreatment. Among all detected metabolites, L-1β-IN-1 showed statistically significant differences in 11 metabolites compared to the fentanyl group and returned 29 metabolites (94%) to non-statistically significant levels compared to control. However, CBD showed 3 metabolites with statistically significant differences compared to the fentanyl group and normalized 25 metabolites (81%) to statistically insignificant levels compared to control (Supplementary Figure S2). This pattern is consistent with the sPLS-DA plot (Figure 3B), in which the L-1β-IN-1 cluster was closer to the control cluster, indicating broader metabolome normalization at half the CBD dosage in these samples. Although both pretreatments shifted the fentanyl-altered metabolomic profile towards the control, L-1β-IN-1 data showed a larger and statistically distinct subset of metabolites, including butanedioic acid, Myo-Inositol, alpha-ketoglutaric acid, and norleucine, whereas CBD normalization was driven by only three statistically significant metabolites against a broader background of non-significant shifts.

The broader anti-inflammatory properties previously reported for CBD are consistent with several findings suggesting its modulatory effects on neuroinflammation via suppression of IL-1β and iNOS expression [23,46]. However, the broader metabolomic modulation observed with L-1β-IN-1 may reflect a related or distinct effect on the fentanyl-induced metabolomic changes. One possible mechanistic explanation, which remains to be tested, for the stronger effects observed with the synthetic analog is that it blocks IL-1β and limits the downstream inflammatory cascade linked to glutamate release, consistent with reports showing that IL-1β upregulates glutamate production through increased glutaminase activity [47]. While the L-1β-IN-1 effects on glutamate itself did not reach statistical significance in the presented dataset, it significantly normalized several metabolites that are metabolically or enzymatically coupled to glutamate regulation and among the most tightly connected nodes in the fentanyl group correlation network, including alpha-ketoglutaric acid, aspartic acid, N-acetyl-L-aspartic acid, and butanedioic acid. Glutamate itself shows a strong positive correlation to these same TCA cycle intermediates, consistent with previous findings that glutamate is a key alternative substrate for supplying the TCA cycle under mitochondrial stress [48]. We note that this correlation network was constructed from the fentanyl group only and therefore describes co-regulated changes among fentanyl-disrupted metabolites; therefore, it does not provide definitive evidence of the pretreatment effects on this network. Rather, its value relies on identifying which of the fentanyl-disrupted metabolites are functionally coupled. The overlap between these tightly connected network nodes and the significant normalizing effect of L-1β-IN-1 suggests that its effect is not limited to isolated metabolites but extends across a functionally interconnected node of glutamate/TCA cycle metabolism disrupted by fentanyl. We emphasize that this remains an association drawn from combining two different analyses, rather than a direct statistically tested comparison of network structure between the fentanyl and L-1β-IN-1 groups, and that our study did not directly measure IL-1β levels and systematically evaluate the effects of L-1β-IN-1 on glutamate and its coupled metabolites.

In addition, among these interconnected nodes, NAA, a widely used marker of neuronal and mitochondrial health, occupied a central position in the network, showing statistically significant correlations with 18 of the detected metabolites and its strongest association with aspartic acid (Supplementary Figure S1). Its normalization by L-1β-IN-1 is consistent with, but not direct evidence of, a neuroprotective effect and remains an important direction for further histological or functional assays to address whether L-1β-IN-1 broad metabolomic normalization translates into actual protection of neurons against fentanyl-induced damage.

Along with the change in metabolic signatures after chronic fentanyl administration, we performed open field studies that are important for validating whether the observed changes in neurometabolic signatures are associated with disruption in general behavioral effects following fentanyl overdose and its modulation by chronic treatment with fentanyl alone or in combination with CBD, or L-1β-IN-1. Observations from these studies show a significant increase in locomotor activity after fentanyl overdose. This is indicative of the neurobehavioral changes that are in line with previous observations of acute fentanyl exposure in mice [40]. Challenging the co-treatment group with 1 mg/kg fentanyl showed an increase in activity during the test session in the CBD group, whereas the L-1β-IN-1 group failed to produce a significant enhancement in the locomotor counts during the test session. While these observations indicate differences in behavioral outcomes between CBD and L-1β-IN-1, it is difficult to speculate on the exact explanation, and additional studies are required to clarify whether CBD and L-1β-IN-1 exert pharmacokinetic (PK) or pharmacodynamic (PD) interactions with chronic fentanyl that underlie these behavioral outcomes. As all behavioral and neurometabolic assessments in these studies were conducted exclusively in male mice, it remains to be determined whether these findings generalize to a larger sample size and female mice, given documented sex differences in CBD pharmacology.

Within the context of clinical translatability, there are several concerns regarding adverse events and drug interactions between the opioids and cannabinoids, despite the clinical and preclinical evidence reporting the beneficial effects of CBD in modulation of opioid reward or withdrawal effects, and protection from respiratory depression [22,24,49]. For example, CBD has been shown to increase liver function test values and requires further study to assess its impact on suicidal ideation [50]. Similarly, future systematic dose titration studies are needed to compare the efficacy of L-1β-IN-1 in modulating fentanyl PK, particularly its potential for CYP450 isoform-mediated drug interactions. Additionally, respiratory safety studies must be conducted to evaluate the safety of modulating the cannabinoid system with L-1β-IN-1 as a possible therapeutic strategy in OUD, including confirmation of whether IL-1β Inhibition is the mechanism responsible for any observed effects.

## 4. Materials and Methods

i. Drugs and Chemicals

Fentanyl Citrate was obtained from the Ministry of Health in Saudi Arabia. Cannabidiol (CBD) and L-1β-IN-1 were purchased from LGC GmbH, Wesel, Germany, and MedChemExpress, Monmouth Junction, NJ, USA, respectively.

Fentanyl was dissolved in distilled water to form the drinking solution with (*v*/*v*) concentrations of 0.01 and 0.03 mg/mL. The doses of each drug used in these experiments are expressed as the free base. In pretreatment studies with either CBD or L-1β-IN-1, all drugs and controls were administered via intraperitoneal (i.p.) injections in volumes of ≤10 mL/kg. DMSO was used to prepare the stock solutions of CBD and L-1β-IN-1. Then adequate volumes of saline were added to the solution to prepare 10 mg/kg CBD and 5 or 25 mg/kg L-1β-IN-1. This was done to ensure that the DMSO was below 10% and had no impact on the conducted studies [51]. Saline i.p. injections were used in the control groups.

ii. Subjects

Naïve male C57BL/6 mice weighing 25–35 g served as subjects and were purchased from the Research Animal Care Facility at King Saud University (KSU). Initially, in each group, *N* = 6–8 mice were used in all behavioral experiments to provide sufficient statistical power to detect statistically significant differences. All mice were group-housed in a temperature- and humidity-controlled vivarium with a 12-h light/dark cycle (lights on at 7:00 AM). Experiments were conducted during the light phase between 9:00 AM and 5:00 PM. Mice had continuous access to food and water except during the experiment sessions. All mice were maintained per the guidelines provided by the Guide for the Care and Use of Laboratory Animals [52]. The experimental protocol (no. KSU-SE-25-24) was approved by the Institutional Animal Care and Use Committee at KSU, Riyadh, Saudi Arabia. At the end of the experiment, all mice were briefly exposed to CO_2_, followed by direct decapitation, and the brains were collected and stored at −80 °C.

iii. Two-Bottle-Choice (TBC) Paradigm and Habituation

- Group assignments, behavioral habituation, and weight tracking

**Figure 8:**
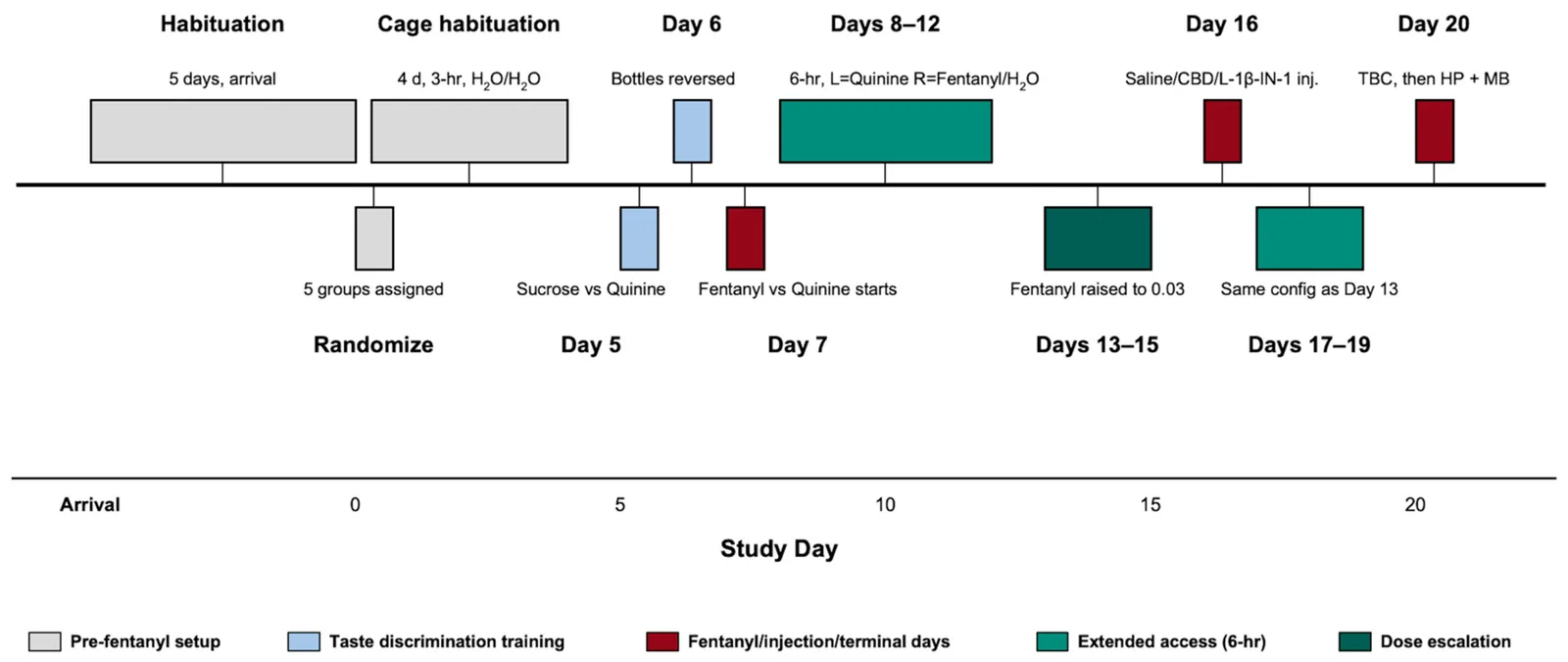
Timeline of the two-bottle-choice (TBC) paradigm from arrival through Day 20. Bar color indicates phase type. The water-only group received water in both bottles throughout the experiments to track the baseline drinking volumes.

Before starting the experiment, the subjects were habituated to the environment for 5 days. Following the habituation period, the mice were weighed and randomly assigned using the RAND function in Excel to assign them to different groups. Five groups were used to assess the effects of cannabidiol (CBD) and L-1β-IN-1 on fentanyl consumption in a two-bottle-choice (TBC) paradigm: 1—Water group, water available in both bottles throughout, serving as a fluid-intake control with no drug or choice component, 2—Fentanyl only, 3—Fentanyl + 10 mg/kg CBD, 4—Fentanyl + 5 mg/kg L-1β-IN-1 and 5—Fentanyl + 25 mg/kg L-1β-IN-1. The selection of pretreatment doses was based on previously published opioid-related studies in C57BL/6 mice [32,53,54]. L-1β-IN-1 (5 and 25 mg/kg) doses were selected based on established anti-inflammatory and IL-1β-inhibitory dosing of CBD and its C-methylated derivatives in mice, where 25 mg/kg produced significant efficacy in vivo [26]. Group sizes, timeline, and behavioral endpoints are detailed in Figure 8.

Afterward, 3-h habituation sessions were conducted daily for 4 days to familiarize the mice with being individually placed in the two-bottle-choice testing cages. During the habituation sessions, each testing cage contained 2 bottles of 15 mL of water (labeled as left and right). At the end of each session, the bottles were weighed and their volumes recorded to determine the baseline water consumption from the left and right bottles. Volume recording was performed by two researchers blinded to the treatment group; raw left/right bottle volumes were subsequently remapped to substance identity (fentanyl, quinine, or water) to account for daily bottle-position assignment, including the position switches.

Throughout the experiment, mice were weighed at least three times per week to monitor body weight changes. Bottle weights and volumes were documented only after the TBC session to measure daily fluid consumption.

- Access Fentanyl Oral Administration Paradigm of Testing

On Day 5, animals were trained to distinguish between flavored solutions. Sucrose and quinine (Q water) solutions were prepared at 0.1% *w*/*v* for the 3-h TBC session. On Day 6, bottle positions were reversed to control for positional bias, and a second 3-h sucrose vs. Q water session was conducted. On Day 7, fentanyl (0.01 mg/mL) was introduced in place of sucrose; animals in the fentanyl-exposed groups were given a 3-h choice between fentanyl and 0.1% *w*/*v* quinine. From Days 8–12, TBC sessions were extended to 6 h per day. The left bottle contained 0.1% *w*/*v* quinine, and the right bottle contained either 0.01 mg/mL fentanyl or water (in group one only). On Days 11 and 12, bottle positions were switched to minimize positional preference. On Days 13–15, the fentanyl concentration was increased (0.03 mg/mL). On Day 16, animals received pretreatment injections of saline, cannabidiol (CBD; 10 mg/kg), or L-1β-IN-1 (5 or 25 mg/kg) prior to the fentanyl drinking session. Fluid intake was measured for the subsequent two days using the same TBC configuration described for Day 16. This procedure was continued through Day 20.

- Rationale for quinine adulteration

Quinine (Q water) was included in the non-fentanyl bottle to control for the intrinsic bitterness of the fentanyl solution. This was based on previously established opioid two-bottle-choice designs to match the taste profile of the drug solution rather than leaving it unadulterated [55]. Particularly, in C57BL/6, opioid TBC preference was strongly influenced by a matched taste comparator where drug solution avoidance occurred with water, but a bitter agent shifted preference toward the drug. Also, there is evidence that oral fentanyl was resistant to quinine adulteration; mice continued responding for fentanyl at quinine levels that suppressed sucrose responding, indicating reinforcement rather than taste-avoidance in these studies [27,56].

- Hot Plate Assay

Nociceptive response was evaluated by a blinded researcher using the hot plate assay. Animals were placed individually on a thermostatically controlled hot plate (Ugo Basile, Gemonio, Italy) maintained at 55 ± 0.5 °C. The latency to the first nociceptive response (paw licking, paw shaking, and jumping) was recorded, with a cutoff time of 10 s to prevent tissue damage. Increased response latency was interpreted as an analgesic effect. First, baselines were recorded. Then, a second experiment was done immediately after the final two-bottle-choice session to test whether the pretreatment with CBD or L-1β-IN-1 modified the fentanyl-induced effects.

- Marble-Burying Test

The marble-burying test was conducted using twenty glass marbles evenly arranged on the surface of a 5-cm layer of bedding in individual test cages. The marbles were positioned superficially and uniformly spaced. Each mouse was placed individually into a test cage and allowed to habituate for 30 min. Subsequently, in test sessions, each mouse was reintroduced into its respective test cage for 30 min. Between testing sessions, all marbles used in the marble-burying test were sterilized using 70% isopropyl alcohol to eliminate residual scent cues from the previously tested mice.

The number of buried marbles was recorded independently by two blinded researchers. The testing criteria were established based on previously published research [57] in which a marble was considered buried if more than half of its surface was covered by bedding. The number of marbles buried by each mouse was used as an index of compulsive-like behavior. According to the predetermined criterion, mice burying 10 or more marbles (≥50%) were classified as exhibiting obsessive-compulsive-like behavior.

iv. The effects of CBD or L-1β-IN-1 in Chronic High Doses of Fentanyl Administration

- Study Design

Animals were randomly allocated to one of the four experimental groups [Control, Fentanyl, +10 mg/kg CBD, and +5 mg/kg L-1β-IN-1] using the RAND function in Excel. Biological samples from *n* = 4–5 brains were used based on previously established metabolomics mass spectrometry procedures [58,59]. The control group received twice-daily injections of intraperitoneal (i.p.) normal saline for 5 days. The remaining groups were given 0.03 mg/kg i.p. fentanyl twice daily, and the dose of fentanyl was increased to 1 mg/kg as an overdose on day 5. The pretreatment paradigm with either CBD or L-1β-IN-1 was to give a single i.p. injection on days 1 and 3, 30 min before fentanyl administration. The mice were euthanized by brief exposure to CO_2_ followed by direct decapitation on day 5, and the brains were immediately collected and stored at −80 °C. The left hemispheres were used for metabolomic profiling, and the right hemispheres were stored for future proinflammatory cytokine analysis.

- Open Field Studies

A comprehensive approach to assessing locomotor activity as a general behavior in mice (*n* = 8) through the open field activity monitoring system was used to evaluate fentanyl-induced hyperlocomotion. First, locomotor baselines were established for all groups before chronic injections. The mice were habituated in the open field chambers for 10 min. Then the test was started, and the mice were allowed to explore for 10 min. During this period, ambulatory locomotor counts were recorded using the Supermex apparatus (Muromachi Kikai, Tokyo, Japan) equipped with a sensor monitor positioned above the testing chamber.

At day 5 after the overdose injections, the mice’s activity was reevaluated to test whether 10 mg/kg CBD or 5 mg/kg L-1β-IN-1 treatment could modify these effects.

- Brain Metabolomics

Untargeted metabolomic analysis was performed on the left hemispheres of the collected brains as described previously [12]. Gas chromatography–mass spectrometry (GC-MS) was utilized for derivatization and metabolic profiling [60,61]. In brief, frozen samples were defrosted under controlled environmental conditions, weighed, and homogenized in methanol to extract the metabolites, and then centrifuged for 5 min at 10,000 rpm at 4 °C. After centrifugation, 200 μL of the supernatant was transferred to a 2 mL GC-MS vial. The samples were then dried using nitrogen. An additional 100 μL of 20 mg/mL methoxyamine hydrochloride was added to the pyridine solution, followed by 10 min of vortex mixing and 16 h of incubation. After the incubation period, the samples were derivatized using BSTFA/TMCS (99/1, *v*/*v*), and 1 μL was injected using a split-mode injection device (split ratio of 1:20). GC-MS was conducted using a PerkinElmer Clarus 600 gas chromatograph coupled to a Clarus 600 T mass spectrometer (PerkinElmer, Shelton, CT, USA). Data were acquired and processed using TurboMass software version 6.1.2.2058.

v. Statistical Analysis

Statistical analyses were performed using Prism GraphPad software 10 (GraphPad Software, San Diego, CA, USA). Behavioral and metabolomics experiments involving multiple groups were analyzed using one- or two-way analysis of variance (ANOVA), followed by either Tukey’s or Dunnett’s post hoc tests to evaluate the differences for multiple comparisons. All data are presented as mean ± standard error of the mean (SEM), and statistical significance was set at *p* < 0.05. Animals that died or reached a humane endpoint were excluded from analysis; this occurred in the 25 mg/kg L-1β-IN-1 group only.

Fentanyl preference was assessed using a two-bottle-choice paradigm. The preference score was calculated as the proportion of total fluid intake consumed as fentanyl solution using the following formula:

*Preference score* = *fentanyl intake*/(*fentanyl intake* + *Q water intake*).

Preference values greater than 0.5 were interpreted as fentanyl preference.

Thermal nociception as an estimate for the analgesic effects was expressed as the percentage of maximum possible effect (%MPE), calculated as:

%*MPE* = 100 × (*test latency* − *baseline latency*)/(*cutoff latency* − *baseline latency*)

The outcome of marble burying was presented as the percentage of marbles buried (%MB), calculated as the number of marbles buried divided by the total number of marbles, multiplied by 100.

To determine the relative abundance of each metabolite, individual peak areas were divided by the mean peak area of the corresponding metabolite within the control group. This normalization process allowed for a direct comparison of metabolite levels across all experimental samples relative to the control baseline.

Network analysis, enrichment analysis, and their statistical tests represent the effect of the fentanyl group alone on the metabolic profile and were performed using MetaboAnalyst 6.0 and Python 3.13 (SciPy 1.16.3, pandas 2.2.3, NetworkX 3.6.1) in Google Colaboratory. Brain hemispheres identified as statistical outliers (>2 SD from the group mean) in the metabolomic dataset were excluded from the analysis, accounting for the reduction from *n* = 5 to *n* = 4. Statistical significance was set at *p* < 0.05.

## 5. Conclusions

Previous work established the in vitro and in vivo efficacy of L-1β-IN-1 as a potent IL-1β inhibitor in inflammatory models; the present study provides proof of concept for its behavioral and neurochemical modulatory role against fentanyl effects in male mice, rather than mechanistic confirmation that these effects are IL-1β-dependent. These studies provide behavioral evidence that CBD and its synthetic derivative can similarly reduce fentanyl preference in our TBC paradigm. In parallel, the neurometabolic studies revealed differential effects of these compounds on the fentanyl-induced brain neurometabolomic signature. Together, these findings support further evaluation of L-1β-IN-1 as a candidate pharmacological tool for OUD research.

## Data Availability

The original contributions presented in this study are included in the article/Supplementary Materials. Further inquiries can be directed to the corresponding author.

## Supplementary Materials

The following supporting information can be downloaded at: https://www.mdpi.com/article/10.3390/molecules31193463/s1. Table S1: Statistical analyses of all metabolites listed in Figure 5 and Figure 6; Figure S1: Differential metabolomic modulation by CBD and L-1β-IN-1 relative to fentanyl and control. Number of brain metabolites showing significant modulation relative to the fentanyl group or no significant difference from the control group following treatment with CBD (10 mg/kg) or L-1β-IN-1 (5 mg/kg). Of the 31 detected metabolites, CBD significantly modulated 3 metabolites (10%) relative to fentanyl and showed no significant difference from control for 25 metabolites (81%), whereas L-1β-IN-1 significantly modulated 11 metabolites (35%) relative to fentanyl and showed no significant difference from control for 29 metabolites (94%). (B) Heatmap showing pairwise statistical comparisons for each detected metabolite across control, fentanyl, CBD, and L-1β-IN-1 groups. Comparisons are presented as control vs. fentanyl, fentanyl vs. CBD, fentanyl vs. L-1β-IN-1, control vs. CBD, and control vs. L-1β-IN-1. Metabolites are ranked according to the *p*-value for the fentanyl vs. L-1β-IN-1 comparison. Statistical significance was determined using Tukey HSD pairwise comparisons. ns, *p* ≥ 0.05; *, *p* < 0.05; **, *p* < 0.01; ***, *p* < 0.001; Figure S2: Significance of pairwise correlations among detected brain metabolites. Hierarchically clustered heatmap illustrating the statistical significance of Pearson pairwise correlations among the 31 detected brain metabolites. Each cell represents the *p*-value associated with the correlation between the corresponding metabolite pair. The matrix is symmetrically displayed, with the diagonal representing self-correlations. Metabolites are hierarchically clustered according to their correlation patterns. ns, *p* ≥ 0.05; *, *p* < 0.05; **, *p* < 0.01; ***, *p* < 0.001.

## Abbreviations

The following abbreviations are used in this manuscript:

%MB	Percentage of marble buried
%MPE	Percentage of maximum possible effect
BSTFA/TMCS	Bis(trimethylsilyl)trifluoroacetamide + 1% trimethylchlorosilane
CBD	Cannabidiol
CDC	Centers for Disease Control and Prevention
CNS	Central nervous system
DMSO	Dimethyl sulfoxide
IL-1β	Interleukin-1 beta
IL-6	Interleukin-6
iNOS	Inducible nitric oxide synthase
L-1β-IN-1	Synthetic derivative of cannabidiol (CBD)
OUD	Opioid use disorder
TBC	Two-bottle choice
TCA	Tricarboxylic acid cycle
TNF-α	Tumor necrosis factor-alpha
Q water	Water containing 0.1% *w*/*v* quinine
