# Supplementary material for: Impact of Cannabidiol and Its Synthetic Derivative on Fentanyl Consumption and Fentanyl-Induced Neurometabolic Alterations in Mice
Source: Molecules. 2026 Sep 28;31(19):3463. doi: 10.3390/molecules31193463 (PMC13666421; doi:10.3390/molecules31193463)
Supplement: Supplementary file 1 [file molecules-31-03463-s001.zip › molecules-4529547-supplementary.pdf]

Effects of CBD or L-1β-IN-1 on Fentanyl-Driven Brain Metabolite Fluctuations

Supplementary Table S1. Statistical analysis for all the listed metabolites in Figures 5 and 6

Metabolite	Control Mean ± SEM	Fentanyl Mean ± SEM	+10 mg/kg CBD Mean ± SEM	+5 mg/kg L-1β-IN-1 Mean ± SEM	One-way ANOVA	Tukey HSD p-adj					
	Ratio				F ; p values	Control vs Fentanyl	Fentanyl vs +10 CBD	Fentanyl vs +5 L-1β-IN-1	Control vs +10 CBD	Control vs +5 L-1β-IN-1	+10 CBD vs +5 L-1β-IN-1
Alpha-Ketoglutaric Acid	1.00 ± 0.25	3.57 ± 0.57	2.82 ± 0.15	1.18 ± 0.30	12.524 ; 0.0005	0.0012 (**)	0.4682 (ns)	0.0023 (**)	0.0158 (*)	0.9829 (ns)	0.0299 (*)
Butanedioic Acid	1.00 ± 0.45	3.35 ± 0.36	1.34 ± 0.44	0.18 ± 0.05	13.688 ; 0.0004	0.0031 (**)	0.0099 (**)	0.0002 (***)	0.9072 (ns)	0.4150 (ns)	0.1608 (ns)
Lactic Acid	1.00 ± 0.11	2.12 ± 0.42	1.61 ± 0.17	1.28 ± 0.18	3.779; 0.0405	0.0335 (*)	0.4987 (ns)	0.1318 (ns)	0.3389 (ns)	0.8493 (ns)	0.7799 (ns)
Glyceric Acid	1.00 ± 0.25	3.24 ± 0.55	1.52 ± 0.32	0.88 ± 0.27	8.733; 0.0024	0.0050 (**)	0.0285 (*)	0.0033 (**)	0.7508 (ns)	0.9951 (ns)	0.6154 (ns)
Oxalic Acid	1.00 ± 0.36	0.03 ± 0.01	0.03 ± 0.01	0.01 ± 0.00	7.275; 0.0049	0.0122 (*)	1.0000 (ns)	0.9998 (ns)	0.0116 (*)	0.0106 (*)	0.9999 (ns)
2,3,4-Trihydroxybutanoic Acid	1.00 ± 0.08	4.09 ± 0.50	2.41 ± 0.38	2.06 ± 0.51	9.854; 0.0015	0.0009 (***)	0.0550 (ns)	0.0191 (*)	0.1229 (ns)	0.3086 (ns)	0.9275 (ns)
Phosphoric Acid	1.00 ± 0.49	3.72 ± 0.59	2.62 ± 0.40	1.25 ± 0.67	5.377; 0.0141	0.0191 (*)	0.5067 (ns)	0.0337 (*)	0.2100 (ns)	0.9874 (ns)	0.3344 (ns)
Timonacic Acid	1.00 ± 0.32	1.45 ± 0.21	1.15 ± 0.23	0.72 ± 0.28	1.312; 0.3158	0.6372 (ns)	0.8473 (ns)	0.2612 (ns)	0.9791 (ns)	0.8779 (ns)	0.6778 (ns)
L-Glutamic Acid	1.00 ± 0.68	7.75 ± 1.10	4.86 ± 1.73	3.07 ± 1.85	4.037; 0.0337	0.0256 (*)	0.5023 (ns)	0.1471 (ns)	0.2720 (ns)	0.7359 (ns)	0.8117 (ns)
Beta-Glutamic Acid	1.00 ± 0.35	4.28 ± 0.63	4.37 ± 0.26	2.46 ± 0.59	11.147 ; 0.0009	0.0021 (**)	0.9993 (ns)	0.0840 (ns)	0.0017 (**)	0.1950 (ns)	0.0682 (ns)
Pyroglutamic Acid	1.00 ± 0.47	2.50 ± 0.79	1.45 ± 0.57	0.33 ± 0.17	2.794; 0.0858	0.2613 (ns)	0.5418 (ns)	0.0651 (ns)	0.9367 (ns)	0.8199 (ns)	0.4954 (ns)
Aspartic Acid	1.00 ± 0.51	4.96 ± 1.54	2.15 ± 0.71	0.58 ± 0.31	4.823; 0.0199	0.0389 (*)	0.1751 (ns)	0.0217 (*)	0.8039 (ns)	0.9865 (ns)	0.6170 (ns)

Effects of CBD or L-1β-IN-1 on Fentanyl-Driven Brain Metabolite Fluctuations

N-Acetyl-L-Aspartic Acid	1.00 ± 0.52	6.81 ± 2.08	3.77 ± 1.15	0.42 ± 0.14	5.793; 0.011	0.0247 (*)	0.3350 (ns)	0.0137 (*)	0.4101 (ns)	0.9863 (ns)	0.2612 (ns)
L-Asparagine	1.00 ± 0.25	6.56 ± 1.12	4.82 ± 1.15	1.25 ± 0.78	9.189; 0.002	0.0044 (**)	0.5392 (ns)	0.0062 (**)	0.0478 (*)	0.9971 (ns)	0.0672 (ns)
N-Acetyl Glycine	1.00 ± 0.51	4.53 ± 1.40	1.48 ± 0.57	1.40 ± 0.55	3.761; 0.041	0.0503 (ns)	0.0997 (ns)	0.0886 (ns)	0.9768 (ns)	0.9867 (ns)	0.9999 (ns)
L-Serine	1.00 ± 0.50	0.23 ± 0.03	0.19 ± 0.01	1.07 ± 0.92	0.824; 0.5056	0.7327 (ns)	1.0000 (ns)	0.6761 (ns)	0.7061 (ns)	0.9996 (ns)	0.6488 (ns)
L-Threonine	1.00 ± 0.12	3.99 ± 0.41	2.96 ± 0.71	1.40 ± 0.83	5.627; 0.0121	0.0162 (*)	0.6108 (ns)	0.0380 (*)	0.1372 (ns)	0.9605 (ns)	0.2866 (ns)
Norleucine	1.00 ± 0.24	6.76 ± 0.77	3.65 ± 0.22	2.98 ± 0.92	14.785; 0.0002	0.0001 (***)	0.0184 (*)	0.0049 (**)	0.0464 (*)	0.1638 (ns)	0.8720 (ns)
DL-Valine	1.00 ± 0.85	4.19 ± 1.37	2.84 ± 0.65	0.78 ± 0.44	3.233; 0.0607	0.1087 (ns)	0.7192 (ns)	0.0813 (ns)	0.4946 (ns)	0.9980 (ns)	0.4020 (ns)
Arachidonic Acid	1.00 ± 0.42	1.53 ± 0.62	0.57 ± 0.15	3.27 ± 1.26	2.579; 0.1022	0.9549 (ns)	0.7921 (ns)	0.3833 (ns)	0.9749 (ns)	0.1859 (ns)	0.0955 (ns)
Myo-Inositol	1.00 ± 0.33	5.49 ± 0.58	3.89 ± 0.25	2.22 ± 0.43	22.025; <0.0001	0.0000 (***)	0.0771 (ns)	0.0006 (***)	0.0018 (**)	0.2191 (ns)	0.0638 (ns)
D-Glucitol	1.00 ± 0.22	2.43 ± 0.25	2.27 ± 0.33	1.81 ± 0.39	4.375; 0.0267	0.0278 (*)	0.9794 (ns)	0.5035 (ns)	0.0545 (ns)	0.2897 (ns)	0.7265 (ns)
Stearic Acid	1.00 ± 0.45	2.93 ± 0.05	1.93 ± 0.12	2.35 ± 0.30	8.62; 0.0025	0.0017 (**)	0.1019 (ns)	0.4807 (ns)	0.1342 (ns)	0.0215 (*)	0.7113 (ns)

Effects of CBD or L-1β-IN-1 on Fentanyl-Driven Brain Metabolite Fluctuations

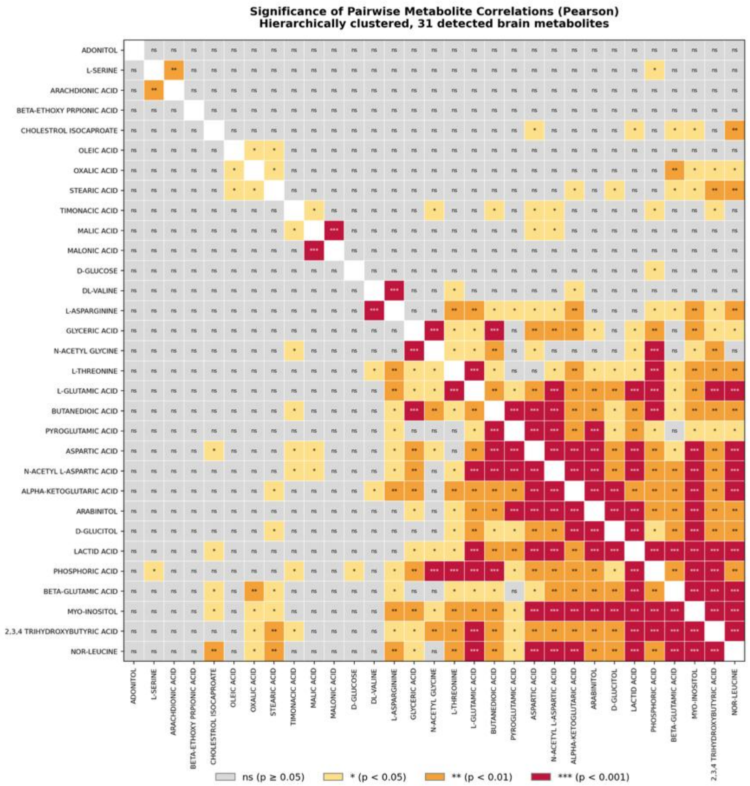

Supplementary Figure S1. Differential metabolomic modulation by CBD and L-1β-IN-1 relative to fentanyl and control. (A) Number of brain metabolites showing significant modulation relative to the fentanyl group or no significant difference from the control group following treatment with CBD (10 mg/kg) or L-1β-IN-1 (5 mg/kg). Of the 31 detected metabolites, CBD significantly modulated 3 metabolites (10%) relative to fentanyl and showed no significant difference from control for 25 metabolites (81%), whereas L-1β-IN-1 significantly modulated 11 metabolites (35%) relative to fentanyl and showed no significant difference from control for 29 metabolites (94%). **(B)** Heatmap showing pairwise statistical comparisons for each detected metabolite across control, fentanyl, CBD, and L-1β-IN-1 groups. Comparisons are presented as control vs. fentanyl, fentanyl vs. CBD, fentanyl vs. L-1β-IN-1, control vs. CBD, and control vs. L-1β-IN-1. Metabolites are ranked according to the p-value for the fentanyl vs. L-1β-IN-1 comparison. Statistical significance was determined using Tukey HSD pairwise comparisons. ns, $p \geq 0.05$; *, $p < 0.05$; **, $p < 0.01$; ***, $p < 0.001$.

Effects of CBD or L-1β-IN-1 on Fentanyl-Driven Brain Metabolite Fluctuations

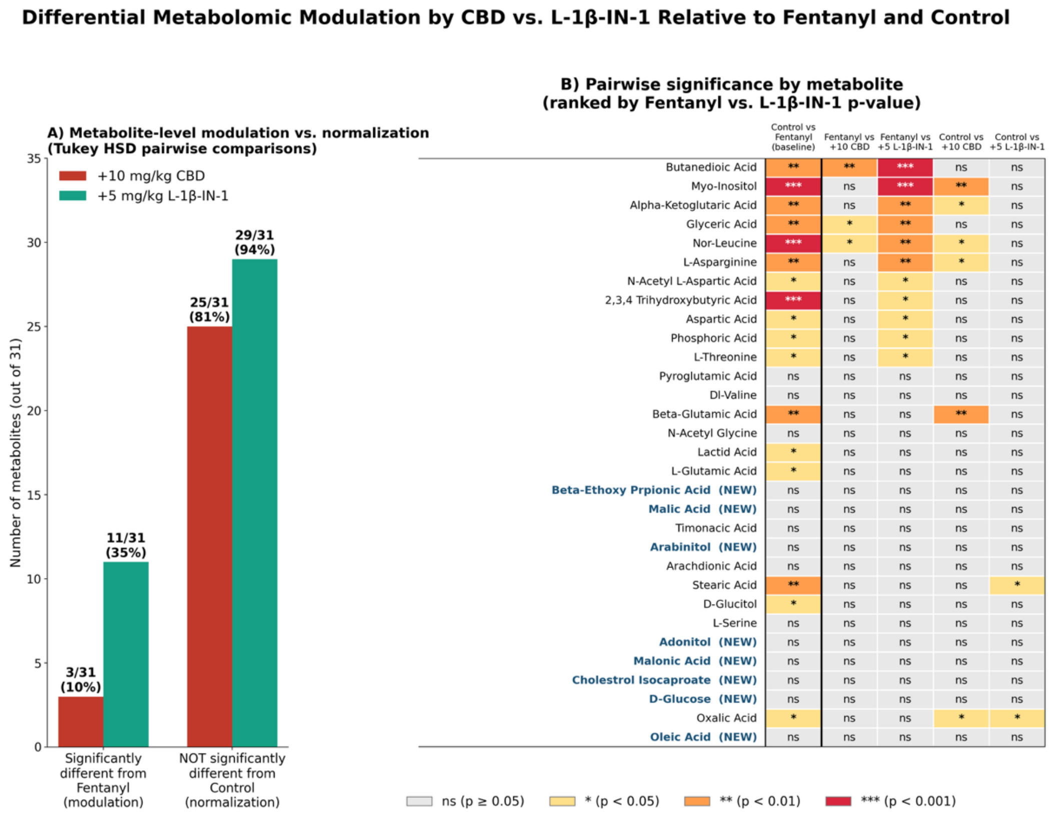

Supplementary Figure S2. Significance of pairwise correlations among detected brain metabolites. Hierarchically clustered heatmap illustrating the statistical significance of Pearson pairwise correlations among the 31 detected brain metabolites. Each cell represents the p-value associated with the correlation between the corresponding metabolite pair. The matrix is symmetrically displayed, with the diagonal representing self-correlations. Metabolites are hierarchically clustered according to their correlation patterns. ns, $p \geq 0.05$; *, $p < 0.05$; **, $p < 0.01$; ***, $p < 0.001$.
